# Subjective effectiveness of ibogaine treatment for problematic opioid consumption: Short- and long-term outcomes and current psychological functioning

**DOI:** 10.1556/2054.01.2017.009

**Published:** 2017-10-17

**Authors:** ALAN K. DAVIS, JOSEPH P. BARSUGLIA, AUSTIN-MARLEY WINDHAM-HERMAN, MARTA LYNCH, MARTIN POLANCO

**Affiliations:** 1 Department of Psychiatry and Behavioral Sciences, Behavioral Pharmacology Research Unit, Johns Hopkins School of Medicine, Baltimore, MD, USA; 2 Crossroads Treatment Center, Rosarito, Mexico; 3 Yale School of Medicine, New Haven, CT, USA

**Keywords:** ibogaine, heroin, prescription opioids, outcomes, effectiveness

## Abstract

**Background and aims:**

Very few studies have reported the effectiveness of ibogaine as a treatment for chronic opioid use. Therefore, this study evaluated the acute subjective effects of ibogaine, outcomes on problematic opioid consumption, and the long-term associations with psychological functioning.

**Methods:**

Using online data collection, 88 patients who received ibogaine treatment in Mexico between 2012 and 2015 completed our survey.

**Results:**

Most participants (72%) had used opioids for at least 4 years and 69% reported daily use. Most (80%) indicated that ibogaine eliminated or drastically reduced withdrawal symptoms. Fifty percent reported that ibogaine reduced opioid craving, some (25%) reporting a reduction in craving lasting at least 3 months. Thirty percent of participants reported never using opioids again following ibogaine treatment. And over one half (54%) of these abstainers had been abstinent for at least 1 year, with 31% abstinent for at least 2 years. At the time of survey, 41% of all participants reported sustained abstinence (>6 months). Although 70% of the total sample reported a relapse following treatment, 48% reported decreased use from pretreatment levels and an additional 11% eventually achieved abstinence. Treatment responders had the lowest rates of depressive and anxious symptoms, the highest levels of subjective well-being and rated their ibogaine treatment as more spiritually meaningful compared with treatment non-responders.

**Conclusion:**

The results suggest that ibogaine is associated with reductions in opioid use, including complete abstinence, and has long-term positive psychological outcomes. Future research should investigate the efficacy of ibogaine treatment using rigorous longitudinal and controlled designs.

## INTRODUCTION

Opioid addiction has developed into a substantial contributor to global disease burden and is one of the largest public health epidemics in the United States (U.S.) and Europe (Degenhardt et al., 2014). Twelve percent of all people with a substance use disorder are addicted to opioids (SAMHSA, 2015) and rates are greater among vulnerable populations, such as U.S. military veterans (Samoylenko et al., 2010) and chronic pain patients (Sehgal, Manchikanti, & Smith, 2012). Moreover, drug overdose is now the primary cause of accidental death in the U.S., with approximately 78 Americans dying every day from an opioid overdose (Center for Disease Control and Prevention, 2016).

Opioid maintenance therapies (OMT) are the current mainline intervention in the U.S. and although there is a broad literature base on their efficacy, these treatments require long-term use and monitoring with potentially hazardous iatrogenic effects (Andersen, Olaussen, Ripel, & Mørland, 2011; Tennant, 2013; Upadhyay et al., 2010). Further, OMT demonstrate mixed efficacy (Ling & Compton, 2005; Nielsen et al., 2016; Veilleux, Colvin, Anderson, York, & Heinz, 2010) as a high percentage of individuals often relapse during or shortly after tapering off of opioid replacements (Stotts, Dodrill, & Kosten, 2009; Weiss et al., 2011). One way to address these problems is to provide access to a single-dose medication that could interrupt/reduce withdrawal and craving for opioids and provide important psychotherapeutic effects to the patient (e.g., insight, motivation to change), thus allowing the opioid user to address the environmental and behavioral problems associated with their consumption of opioids. An example of such a treatment is the use of ibogaine as an opioid detoxification treatment.

### Ibogaine history and evidence for use as a treatment for opioid addiction

Ibogaine is a naturally occurring alkaloid, obtained from the root bark of the African shrub *Tabernanthe iboga*, and is also produced through semi-synthesis of voacangine from the African tropical tree *Voacanga africana*. Iboga was historically used as a medicinal and ceremonial agent in indigenous cultures in West Central Africa to treat fatigue, physical maladies, and as a sacrament in initiation rituals and rites of passage (Fernandez, 1982; Goutarel, Gollnhofer, & Sillans, 1993). The subjective effects of ibogaine are described with several classifications, as a psychedelic, a dissociative, and most precisely as oneirophrenic, or a substance that invokes a dream state without loss of consciousness (Goutarel et al., 1993).

Ibogaine was initially marketed in France in the 1930s as a medical product called *Lamberene* and was used to treat depression, fatigue, and infectious diseases (Goutarel et al., 1993). In the early 1960s, Howard Lotsof identified ibogaine as effective in ameliorating withdrawal and craving from his own heroin addiction (Alper, Beal, & Kaplan, 2001). Since the substance was added to the Controlled Substance Act in 1970, several Phase I/Phase II human clinical trials were developed but were not completed. In 1993, the US FDA granted approval to Dr. Deborah Mash at the University of Miami for a dose-escalation study, which was subsequently suspended in 1995 due to lack of grant support (Alper et al., 2001; Brown, 2013). In 1993–1994, The National Institute on Drug Abuse (NIDA) developed a Phase I investigation to evaluate pharmacokinetic and safety data in a fixed dosage study for cocaine dependence, but they decided not to fund the implementation of the protocol (Alper, 2001; Alper et al., 2001). In two Phase I studies, low doses of noribogaine (the active metabolite of ibogaine, which has a distinct pharmacological profile) was well tolerated (Glue et al., 2015) and showed a trend toward reduction in opioid withdrawal ratings (Glue et al., 2016).

In clinical settings, a typical flood dose ibogaine experience (15–20 mg/kg) results in a session lasting 12–36 hrs and is segmented into different experiential phases or stages. The initial acute phase begins within the first 1–3 hrs and typically consists of vivid waking dreams, which last for 4–8 hrs and are intensified in a dark environment and with the eyes closed. The second phase is evaluative and has an onset between 8 and 20 hrs after initial dosing during which visual imagery diminishes and individuals often report increased levels of intuition, personal insight, and reflection. During the initial two phases, unpleasant effects can include auditory buzzing (tinnitus-like noise), auditory hypersensitivity, ataxia, dissociation, visual tracers, nausea, and vomiting. The final residual phase has an onset 12–24 hrs after the initial dosing and can last from 24 to 72 hrs depending on dosage and metabolic factors. During this last phase, individuals often report reduced need for sleep and feelings of mental clarity and calmness (Alper & Lotsof, 2007).

Despite the legal restrictions on ibogaine in the U.S. and internationally, several clinical outcome studies have been conducted. For example, in a 1999 case series, 33 individuals were treated for opioid detoxification in the U.S. and the Netherlands (Alper, Lotsof, Frenken, Luciano, & Bastiaans, 1999). Relief of withdrawal symptoms was rapid – within 1–3 hrs of administration. Full resolution of opioid withdrawal symptoms was achieved within 34 hrs. Participants did not exhibit drug-seeking behavior within 24 hrs, which was sustained for 72 hrs following treatment in 75% of patients. In another study, Mash et al. (2001) conducted an open-label prospective evaluation of ibogaine in St. Kitts, West Indies with 32 patients diagnosed with a severe opioid use disorder (OUD). Physician ratings indicated the resolution of withdrawal signs and symptoms at 12, 24, and 36 hrs following ibogaine administration. Self-reports of withdrawal symptoms were also significantly reduced from pre-ibogaine ratings. These results suggested ibogaine was an effective treatment for opiate withdrawal. Furthermore, scores of depression and opioid cravings remained reduced 1 month following treatment.

Yet another study (Bastiaans, 2004) evaluated the long-term effects of ibogaine treatment on drug use and on the long-term medical, psychological, social, and legal outcomes among a sample comprised primarily of opioid users (87%) using a web-based survey. After long-term follow-up in this group, 24% (5 out of 21) quit using all substances with an average drug-free period of 24 months following treatment. An additional 33% of the sample continued to use their primary substance but decreased the amount used. Secondary analyses indicated approximately 60% of subjects reported an improvement in their medical condition, 88% reported improvement in relationships with significant others, 92% reported improvement in anxiety, and 100% reported improvement in depression.

Although these observational and outcome studies on ibogaine suggest that it is an effective compound for treating OUDs because it rapidly mitigates withdrawal symptoms and cravings (Alper et al., 1999; Bastiaans, 2004; Mash et al., 2001), the small sample sizes of these studies combined with the limited long-term evidence for effectiveness restrict our understanding of the benefits of this medicine. Therefore, the current observational study aimed to address this key question by evaluating whether ibogaine treatment was associated with short- and long-term opioid use-reduction and abstinence (up to 3 years posttreatment), and current psychological functioning among a larger sample of patients who received treatment for problematic opioid use from 2012 to 2015 at a treatment facility in Mexico.

## METHOD

### Recruitment procedure

To recruit individuals who had received ibogaine treatment for problematic opioid consumption, we obtained a contact list from the medical director at Crossroads Treatment Center (Crossroads), an ibogaine-assisted detoxification program for individuals with opioid and other substance use disorders. This contact list included 336 individuals who received ibogaine treatment at Crossroads between 2012 and 2015; however, only 285 had active e-mail addresses and/or telephone numbers. Following approval from an independent Institutional Review Board (Solutions IRB; #00008523), we then sent an e-mail (with follow-up reminders at biweekly intervals for 4 months) asking them to participate in an anonymous, web-based survey regarding his or her experiences with, and effectiveness of, ibogaine treatment. Each e-mail provided a brief description of the purpose of this study, the benefits of participating, and a hyperlink that individuals could click if they were interested in participating. In order to meet inclusion criteria for the study, participants had to (a) have received ibogaine treatment at Crossroads between 2012 and 2015, (b) be able to complete an online questionnaire, (c) be at least 18 years old, and (d) be able to read, write, and speak English fluently. As an incentive, and as a way to “pay it forward” for participating in the survey, we donated a total of $500 ($10/participant; up to $500) to the Global Ibogaine Therapy Alliance.

During the recruitment period (August–December 2015), 285 people were contacted by study personnel. Of these, 134 people viewed the informed consent document, consented to participate, and began completing the study materials. However, 33 of these individuals did not complete all of our main ibogaine treatment experience questionnaires and thus were excluded. Of the remaining 101 individuals, 13 had sought ibogaine treatment as a way to treat non-opioid substance problems (e.g., alcohol, amphetamines, and cocaine) and thus were excluded in the present analysis. The final sample was comprised of the remaining 88 participants.

### Treatment setting and content

All participants received treatment at Crossroads on a fee-for-service basis. The ibogaine-assisted detoxification occurred in a residential setting and the duration of treatment was 1 week. Crossroads admits men and women aged 18–60 years. Individuals are excluded from treatment with severe psychiatric conditions including current or past psychotic spectrum disorders, bipolar I disorder, current eating disorders, or symptoms of impaired reality testing or disorganized thinking. Medical exclusions for treatment include prolonged QTc interval, history of heart disease, pulmonary embolism, deep vein thrombosis, severe respiratory conditions, such as emphysema or chronic obstructive pulmonary disease, obesity, gastrointestinal disorders, such as Crohn’s disease or irritable bowel syndrome, chronic infectious diseases, cerebellar dysfunction, delirium, organic brain disease or history of severe traumatic brain injury, epilepsy, current pregnancy, and abnormal electrolytes or impaired hepatic or renal function. Patients are also excluded from treatment if they have used alcohol, amphetamines, cocaine, or psychiatric medications in the prior week or have used long-acting opioids, such as buprenorphine or methadone in the 4 weeks prior to treatment.

For most, their primary substance use problem is related to heroin or prescription opioid use. Prior to treatment at the clinic, applicants undergo a physical examination onsite with one of the staff physicians. This exam includes a history and physical, 12-lead electrocardiogram, drug testing, complete physical, and a complete blood count with differential and metabolic panel. The treatment consists of administration of ibogaine hydrochloride (*Voacanga*-derived) imported from a Canadian company, Phytostan Enterprises, Inc. and certified under Good Manufacturing Practice (GMP) guidelines. Dosing ranges between 15 mg/kg and ±5 mg/kg, depending on weight and severity of polysubstance use. All patients received live cardiac monitoring, intravenous saline and electrolytes, and medical monitoring throughout treatment followed by a short residential stay that includes psychological support and aftercare planning. Crossroads has board-certified physicians who specialize in emergency medicine and para-medics on site at all times while patients are in residence.

### Measures

#### Opioid consumption before and after ibogaine treatment

We developed these items to assess the primary substance for which participants sought ibogaine treatment (i.e., prescription opioids or heroin), whether they had a secondary substance for which they were also seeking treatment, how many years they had consumed their primary substance prior to treatment, the number of days they had used their primary substance in the month leading up to treatment, whether their use of opioids following ibogaine treatment had increased, decreased, stayed the same, or whether they had been abstinent since treatment, and whether they had consumed any opioids in the 6 months prior to the study.

#### Subjective effectiveness of ibogaine treatment

We developed these items to assess participants’ posttreatment craving, posttreatment psychological well-being and mood, subjective effectiveness of treatment, whether they would make the same treatment selection, and how this treatment compared with other treatments.

#### Acute subjective effects of ibogaine

We developed these items using a rational approach based upon the authors’ shared knowledge, literature review, and commonly reported patient experiences. We developed these pilot items to assess the variety of acute subjective effects that one might experience after ingesting ibogaine as a treatment for problematic substance use (e.g., I gained insightful knowledge about myself, I gained insight into the causes or reasons for my addiction, I experienced physical discomfort) as there were no such validated scales available in the literature. Participants were asked to rate his or her agreement with each item on a scale from −2 (*strongly disagree*) to 2 (*strongly agree*). In addition to these items, we asked two questions about the spiritual and personal meaning of one’s ibogaine experience. Participants were asked to rate how meaningful his or her ibogaine experience was on a scale from 1 (*not spiritual or not personally meaningful*) to 7 (*the most spiritual... or the most personally meaningful*).

#### Treatment history

We developed these items to assess the opioid replacement therapies (i.e., suboxone, subutex, methadone, and morphine) and psychological/social treatments (e.g., residential, inpatient detoxification, 12-step groups, and peer support), participants had received prior to receiving ibogaine treatment.

#### Depression, Anxiety, and Stress Scale (DASS-21)

We included this 21-item scale (Lovibond & Lovibond, 1995) to assess the core negative emotional experiences of depression, anxiety, and stress that participants were experiencing during the week prior to the study. The DASS-21 is comprised of three subscales: depression, anxiety, and stress. There are seven items in each subscale and participants are asked to respond to each item on the following scale: 0 (*never*), 1 (*sometimes*), 2 (*often*), 3 (*almost always*). Internal consistency reliability was .91 for the depression subscale, .80 for the anxiety subscale, and .87 for the stress subscale.

#### Satisfaction With Life Survey (SWLS)

We included this 5-item measure (Pavot & Diener, 2008) to assess participants’ general satisfaction with life at the time of the study. Participants were asked to rate their agreement with each item on a scale from −3 (*strongly disagree*) to 3 (*strongly agree*). Internal consistency in the current sample was .91.

#### Demographics

This section of the survey evaluated basic demographic data including age, gender, sexual orientation, ethnicity, and relationship status.

### Data analysis plan

We began by conducting frequency counts of demographic, substance use, and treatment history variables using the entire sample (*n* = 88). Next, we split the sample into two subgroups based on whether they had a positive outcome (i.e., never used primary substance again, decreased use) or negative outcome (i.e., no change in use, use increased) following ibogaine treatment. Using treatment response as an independent variable, we then conducted a series of chi-square and Fisher’s exact analyses with two-proportion *z*-tests, to evaluate whether there were differences in demographic, substance use, and treatment history variables between these treatment response subgroups. Next, using chi-square analyses with two-proportion *z*-tests, and *t*-test analyses, we evaluated whether there were differences in pre- and posttreatment substance use problems, reported acute subjective effects of ibogaine, and current psychological functioning and subjective well-being between these subgroups. All analyses were conducted using SPSS version 23 (IBM Corp., New York, NY, USA).

## RESULTS

### Characteristics of sample and pretreatment substance use and treatment history

As the examination of Table 1 reveals, approximately three fourths (73%) of participants were male, one half (50%) were between the ages of 18 and 34, and 89% identified as White/Caucasian. Over one half (59%) of participants indicated that they had received detoxification with ibogaine at least 1 year prior to the survey, and almost equal proportions of participants indicated that they sought treatment for problematic heroin (51%) or prescription opioid (49%) consumption. Overall, most participants (72%) had been using their primary substance (heroin or prescription opioids) for 4 or more years, and almost one fourth (21%) had been using these substances for 10 or more years prior to ibogaine treatment. In addition, approximately two thirds (69%) had used their primary substance for 30 out of 30 days in the month prior to treatment. See Table 1 for further demographic and substance use/treatment history information.

### Subjective effectiveness of ibogaine treatment for problematic opioid consumption

The majority of the sample (61%) indicated that ibogaine treatment was “Very effective,” and 85% indicated that, looking back, they would have made the same decision to engage in this treatment. Almost three fourths of the sample (71%) indicated that ibogaine treatment was “Much better” compared with other treatments they had tried. Following treatment, almost one half (43%) indicated that they experienced an increase in their mood or psychological well-being lasting 1 month or longer (with 10% reporting increases in mood lasting for more than 5 months). In addition, one half (50%) of the sample indicated that they experienced a reduction in craving for opioids lasting at least 1 week, and 25% of the sample experienced a reduction in craving lasting 3 months or more.

These perceptions of treatment effectiveness notwithstanding, almost one third (30%) of the full sample reported that they *never* returned to using opioids after being treated with ibogaine, and approximately one half (54%) of these abstainers had maintained abstinence for at least 1 year, and almost one third (31%) had been abstinent for 2 or more years. Approximately, one half of the full sample (48%) reported that although they relapsed after treatment, their consumption had decreased from pretreatment levels. In addition, relatively small proportions of participants indicated that they had a neutral or negative response to treatment in terms of their posttreatment substance use. Specifically, 17% reported that their consumption was unchanged after ibogaine treatment and 6% reported that their consumption had increased. Furthermore, at the time of survey, 41% of participants reported that they had been completely abstinent from all opioids for at least 6 months prior to completing the survey, indicating that an additional 11% of participants had eventually achieved abstinence following a relapse on opioids.

### Comparisons of substance use history, ibogaine experiences, and current psychological functioning between treatment response subgroups

As Table 1 reveals, there were no differences in demographic, substance use history, or treatment history variables between those who were considered a treatment responder (i.e., they reported sustained opioid abstinence following treatment or reported that their use decreased) or a non-responder (i.e., they reported that their opioid use had not changed or that it had increased following ibogaine treatment), except that there was a significantly larger proportion of participants in the treatment responder subgroup who reported that their primary substance was prescription opioids (versus heroin) compared with those in the treatment non-responder subgroup (56% vs. 25%, respectively), χ^2^(1, 87) = 5.90, *p* = .021, φ = 0.24.

Next, we evaluated the acute subjective effects of ibogaine (see Table 2). The results from this analysis indicated that over three fourths of participants endorsed the following acute subjective effects of ibogaine: saw visions or visuals (88%), withdrawal symptoms were eliminated or drastically reduced (80%), experienced physical discomfort (74%), saw geometric shapes (68%), and gained insightful knowledge about self (67%). As Table 2 also reveals, there were no differences in reports of acute subjective effects between treatment outcome subgroups except that the responder subgroup agreed more strongly with an item assessing the degree to which their ibogaine experiences contributed to gaining insight into the cause of his or her addiction [*M*_responders_ = 0.41, SD = 1.1 vs. *M*_non-responders_ = −0.47, SD = 1.1, *t*(79) = −3.0, *p* = .004, *d* = .80]. The results also indicated that there were no differences in scores assessing the degree to which one’s ibogaine session was personally meaningful, but those participants in the treatment responder subgroup had a higher mean score on an item assessing the degree to which their ibogaine experiences were spiritually meaningful [*M*_responders_ = 4.4, SD = 1.7 vs. *M*_non-responders_ = 3.2, SD = 2.2, *t*(86) = −2.6, *p* = .011, *d* = .61].

Finally, compared with treatment non-responders, treatment responders had significantly lower mean ratings of depression [*M*_responders_ = 10.6, SD = 8.8 vs. *M*_non-responders_ = 17.6, SD = 9.6, *t*(74) = 2.7, *p* = .008, *d* = .76] and anxiety [*M*_responders_ = 6.2, SD = 6.5 vs. *M*_non-responders_ = 10.8, SD = 7.1, *t*(74) = 2.4, *p* = .018, *d* = .68] at the time of survey. However, there were no statistical differences in subjective level of stress [*M*_responders_ = 12.9, SD = 7.9 vs. *M*_non-responders_ = 17.1, SD = 8.0, *t*(74) = 1.8, *p* = .071], although the effect size was medium, *d* = .53. Finally, those in the treatment responder subgroup had higher mean ratings of subjective well-being [*M*_responders_ = 3.9, SD = 1.7 vs. *M*_non-responders_ = 2.7, SD = 1.4, *t*(86) = −2.8, *p* = .006, *d* = .77] compared with non-responders.

## DISCUSSION

We designed this study to evaluate the short- and long-term outcomes of ibogaine detoxification among individuals with chronic opioid use. Similar to prior studies (Alper et al., 1999; Mash et al., 2001), we found a large proportion (80%) of participants reported that ibogaine greatly reduced or ameliorated withdrawal symptoms during treatment. Also consistent with prior research (Mash et al., 2001), 50% of our sample experienced a reduction in craving lasting for 1 week, and 25% for at least 3 months following treatment. In terms of opioid use outcomes, we found slightly higher rates of abstinence (30%) compared with those found in a sample comprised of primarily opioid users (24%; Bastiaans, 2004). We also found that almost one half of abstainers had maintained abstinence for 1 year and one third for 2 years posttreatment, which is consistent with Basitaans (2004) rates of abstinence (24 months posttreatment). However, we also found that 48% of our sample reported reductions in opioid use following treatment compared with only 33% in Bastiaans’s (2004) study. Finally, although Mash et al. (2001) and Bastiaans (2004) found that patients reported reductions in anxiety and depression following treatment, our findings indicated that long-term levels of depression and anxiety were significantly lower among those with positive outcomes associated with treatment, suggesting that beneficial mood outcomes may be moderated by long-term treatment effectiveness.

Not only are these results consistent with prior research, but this study also extends these findings to a larger sample and included a long-term follow-up. In addition, our findings provide initial evidence suggesting that outcomes were better for individuals who had spiritual experiences and gained insight into the cause of their addiction during their ibogaine session. Because most patients (including those who relapse) experience a reduction in craving and withdrawal symptoms as a result of taking ibogaine, it is possible that these reductions are most beneficial (e.g., lead to sustained abstinence or use-reduction) when coupled with strong spiritual and insightful experiences. Because mystical experiences associated with classic hallucinogens have been shown to mediate positive addiction treatment outcomes (Bogenschutz & Johnson, 2016), our results suggest this may also be the case with ibogaine. Although it would be difficult to predict or control the subjective experience of an ibogaine treatment session, it is possible that interventions could be developed to better prepare an individual for their ibogaine experience, such that insight and spiritual/sacred connection was enhanced during their treatment session. It is also possible that these experiences could be enhanced with interventions aimed at helping to integrate them post-treatment. However, these hypotheses need to be evaluated using rigorous controlled trial designs.

Several methodological limitations may limit the generalizability of our findings. First, we recruited only those participants who received ibogaine treatment from one facility in Mexico, and individuals who received treatment elsewhere may have different outcomes following treatment. Furthermore, it is possible that those participants who were unable to be contacted or who declined to participate had different outcomes following treatment, which may have inflated our estimates of treatment response. Also, the extent to which the effectiveness of this treatment in our sample is representative of the population of patients receiving ibogaine is unknown due to the dearth of studies reported in the empirical literature. As with all retrospective studies, ours is also limited by self-selection, retrospective recall and social desirability biases, and by the fact that one’s perception of their treatment experiences could be impacted by whether that treatment was successful. Finally, the clinical procedures at Crossroads changed approximately half way through the 2012–2015 time frame to include 5-methoxy-N,N-dimethyltryptamine (5-MeO-DMT) following ibogaine treatment in order to help patients integrate and consolidate their ibogaine experiences. Unfortunately, due to an error in our online survey (and because the survey was anonymous), we were unable to determine with 100% accuracy which of the participants had received this second medicine and therefore were unable to formally evaluate whether there were any differences in treatment effectiveness as a result of this change. Therefore, we cannot rule out the possibility that it is the combined effect of both substances that contributed to treatment response. However, as an exploratory analysis, using date of treatment as a grouping variable, we conducted a series of chi-square and *t*-test analyses to evaluate whether there were differences in primary outcomes between those most likely to have received 5-MeO-DMT while at Crossroads versus those who most likely did not receive this medication. There were no statistically significant differences between these groups, thus tempering this limitation.

This study is one of the few to document the outcomes of ibogaine treatment among problematic opioid users. The evidence thus far suggests that ibogaine treatment is a promising treatment alternative for people struggling with opioid addiction. Because one dose of ibogaine seems to work by minimizing opioid withdrawal and craving such that meaningful proportions are able to abstain from or reduce use, such a treatment might have far-reaching effects on individual opioid users and their families, and decrease the strain on communities and the healthcare/addiction treatment systems in the U.S. However, there have been no rigorous randomized controlled trials to date, and any efforts to change the legal status of ibogaine in the U.S. (such as those efforts currently underway in Maryland and Vermont) will be challenging without strong empirical evidence. Therefore, we recommend that future research evaluates the safety of this treatment using a rigorous controlled design. In addition, our findings suggest that treatment response is better when patients report strong spiritual and insightful components of treatment. Providers should consider enhancing such insight and spiritual experiences, perhaps in pre-intervention counseling, or as a post-intervention integrative process in order to maximize treatment response.

## Figures and Tables

**Table 1:** Demographic history, pre-ibogaine substance use patterns, and other treatment history for full sample and each subgroup

Characteristics	Full sample	Treatment responders (*n* = 68)^a^	Treatment non-responders (*n* = 20)^a^	*X*^2^
		
	%	%	%	
Age				4.0
18–24	9	9	10	
25–34	41	37	55	
35–54	39	40	35	
55+	10	13	0	
Gender				3.9
Male	73	68	90	
Female	27	32	10	
Ethnicity				0.0
White/Caucasian	89	85	100	
Non-White/Other	11	15	0	
Education level				4.5
Some high school or HS degree	18	15	30	
Some college or associates degree	51	56	35	
Bachelor’s degree	17	15	25	
Postgraduate degree	14	15	10	
Relationship status				0.6
Single/Divorced	63	60	70	
Married/Partnered	38	40	30	
Time since ibogaine treatment				0.4
Less than 1 year	41	43	35	
1–2 years	33	32	35	
2 years or more	26	25	30	
Primary substance				5.9*
Heroin	51	44^	75^	
Prescription opioids	49	56^	25^	
Secondary substance				12.4
None	46	50	30	
Prescription opioids	15	13	20	
Amphetamines	13	12	15	
Benzodiazepines	7	6	10	
Other	7	7	5	
Alcohol	5	6	0	
Cannabis	3	2	10	
Cocaine	2	3	0	
Heroin	2	2	10	
Food/sugar	1	2	0	
Number of years using primary substance prior to ibogaine treatment				3.4
Less than 1 year	6	4	10	
1–3 years	24	22	30	
4–6 years	30	31	25	
7–9 years	21	19	25	
10 or more years	21	24	10	
Number of days using primary substance in the month prior to ibogaine treatment				3.8
1–9	16	15	20	
10–19	3	2	10	
20–29	11	12	10	
30	69	72	60	
Other treatments tried prior to ibogaine (could check all that apply)				
Inpatient detoxification	53	49	70	2.9
12-step	50	52	45	0.3
Residential	41	38	50	0.9
Peer support	35	38	25	1.2
Psychotherapy	32	32	30	0.0
Psychotropic medications	28	29	25	0.2
Recovery coaching	22	22	20	0.0
Other	16	16	15	0.0
SMART	10	12	5	0.0
Hypnosis	7	9	0	0.0
Type of opioid replacement therapy attempted in the past (could check all that apply)				
Suboxone	66	62	80	2.3
Methadone	42	43	40	0.0
Subutex	31	29	35	0.3
Morphine	13	12	15	0.0
Total number of lifetime ibogaine treatments				
One	78	78	80	2.9
Two	14	16	5	
Three or more	8	6	15	

*Note.* SMART = Self-Management for Addiction Recovery Training.

Totals may not sum to 100% due to rounding. Total number of participants ranged from 67 to 68 per characteristic due to missing data or declining to respond.

^ Values marked with this superscript within a row are significantly different from one another.

a Responder categories were created by combining treatment response subgroups. Participants were considered Responders if they reported that they never returned to using or if their use had decreased and Non-responders were those participants who reported that there was no change in their substance use following treatment or that their use had increased.

* *p* < .05.

**Table 2:** Proportion of participants who indicated they experienced each acute subjective effect of ibogaine, and comparisons of means and standard deviations, of items assessing acute subjective ibogaine experiences between treatment responders and non-responders

Characteristics	Full sample	Treatment responders (*n* = 68)	Treatment non-responders (*n* = 20)	*t*-test statistic
		
	% endorsed^a^	*M* (SD)	*M* (SD)	
I saw visions or visuals	88	1.33 (0.94)	1.53 (0.87)	0.793
My withdrawal symptoms were eliminated or drastically reduced	80	1.05 (1.24)	1.29 (1.05)	0.753
I experienced physical discomfort	74	0.75 (1.20)	0.71 (1.11)	−0.137
I saw geometric shapes	68	0.61 (1.22)	0.88 (1.32)	0.808
I gained insightful knowledge about myself	67	0.81 (1.11)	0.47 (1.28)	−1.092
I experienced something sacred or spiritual	64	0.66 (1.29)	0.18 (1.38)	−1.346
I experienced fear	51	0.11 (1.39)	0.53 (1.07)	1.154
I saw frightening images	49	0.06 (1.46)	0.35 (1.32)	0.744
I experienced a feeling of unity with ultimate reality	48	0.39 (1.29)	−0.24 (1.44)	−1.734
I worked through or released feelings of unhealthy shame or guilt	46	0.30 (1.15)	−0.24 (1.15)	−1.696
I gained insight into the causes or reasons for my addiction	43	0.41 (1.08)	−0.47 (1.13)	−2.951*
I recalled and experienced difficult memories from my past	36	−0.11 (1.27)	−0.29 (1.36)	−0.524
I felt like I was being reborn	32	−0.03 (1.32)	−0.41 (1.37)	−1.047
I gained insight into past trauma in my life	30	−0.03 (1.08)	−0.53 (1.28)	−1.621
I experienced bliss or ecstasy	26	−0.09 (1.28)	−0.59 (1.18)	−1.438

Note.

a This response category was collapsed to include those who selected “Strongly agree” or “Agree” that he or she agreed with statements indicating the experiences each acute subjective effect of ibogaine. Range of scores was −2 to +2 for each item.

* *p* < 0.01.
